# “It was unusual but amazing”: demand creation for PrEP among adolescents’ men who have sex with men (MSM) and transgender women (TGW) in Brazil

**DOI:** 10.1590/0102-311XEN066423

**Published:** 2024-05-17

**Authors:** Anderson Reis de Sousa, Luis Augusto Vasconcelos da Silva, Sandra Assis Brasil, Eliana Miura Zucchi, Dulce Aurélia de Souza Ferraz, Laio Magno, Alexandre Grangeiro, Ines Dourado

**Affiliations:** 1 Escola de Enfermagem, Universidade Federal da Bahia, Salvador, Brasil.; 2 Instituto de Humanidades, Artes e Ciências Professor Milton Santos, Universidade Federal da Bahia, Salvador, Brasil.; 3 Instituto de Saúde Coletiva, Universidade do Estado da Bahia, Salvador, Brasil.; 4 Programa de Pós-graduação em Saúde Coletiva, Universidade Católica de Santos, Santos, Brasil.; 5 Diretoria Regional de Brasília, Fundação Oswaldo Cruz, Brasília, Brasil.; 6 Faculdade de Medicina, Universidade de São Paulo, São Paulo, Brasil.

**Keywords:** HIV, Pre-Exposure Prophylaxis, Adolescent, Health Technology, HIV, Profilaxia Pré-Exposição, Adolescente, Tecnologia em Saúde, VIH, Profilaxia Pre-Exposición, Adolescent, Tecnología para la Salud

## Abstract

This study aimed to analyze the challenges in demand creation for participation in an HIV pre-exposure prophylaxis (PrEP) project in two Brazilian capitals. This qualitative study was conducted with men who have sex with men and transgender women aged 15 to 19 years who lived in two Brazilian state capitals. For this analysis, 27 semi-structured interviews carried out from 2019 to 2020 were evaluated by reflexive thematic content analysis. For participants, PrEP demand creation was essential for their interaction, mediation, bonding, and attachment and proved effective for PrEP acceptability and adherence. Adolescents’ narratives showed that the strategies promoted HIV combination prevention, opened up opportunities for recruitment meetings, helped to negotiate with and convince individuals to use PrEP, strengthened peer education, and evoked a feeling of “being with” and “walking together” despite the challenges. Face-to-face or online interactions using social technologies played a crucial role in recruiting adolescents for the project, expanding knowledge on PrEP and other combination prevention strategies and access to health services and self-care.

## Resumo

Este estudo analisou desafios na criação de demanda para participação em um projeto de profilaxia pré-exposição (PrEP) ao HIV em duas capitais brasileiras. Trata-se de um estudo qualitativo realizado com homens que fazem sexo com homens e mulheres transgênero de 15 a 19 anos residentes de duas capitais brasileiras. Para esta análise, foram avaliadas 27 entrevistas semiestruturadas realizadas entre 2019 e 2020 com análise temática de conteúdo reflexiva. Para os participantes, a criação de demanda por PrEP foi essencial para o processo de interação, mediação, vínculo e apego e mostrou-se eficaz para a aceitabilidade e adesão à PrEP. As narrativas dos adolescentes mostraram que as estratégias promoveram a prevenção combinada do HIV, abriram oportunidades para reuniões de recrutamento, ajudaram a negociar e convencer os indivíduos a usar a PrEP, fortaleceram a educação entre pares e evocaram um sentimento de “estar com” e “caminhar juntos”, apesar dos desafios. As interações, sejam presenciais ou *online*, com o uso de tecnologias sociais, desempenharam um papel crucial no recrutamento de adolescentes para o projeto, na ampliação do conhecimento sobre PrEP e demais estratégias combinadas de prevenção, e no acesso a serviços de saúde e autocuidado.

## Resumen

Este estudio analizó los desafíos para crear demanda para la participación en un proyecto de profilaxis pre-exposición (PrEP) al VIH en dos capitales brasileñas. Se trata de un estudio cualitativo realizado con hombres que tienen sexo con hombres y mujeres transgénero de 15 a 19 años residentes de dos capitales brasileñas. Para este análisis, se evaluaron 27 entrevistas semiestructuradas realizadas entre 2019 y 2020 con un análisis temático de contenido reflexivo. Para los participantes, crear la demanda por PrEP fue fundamental para el proceso de interacción, mediación, vínculo y apego y demostró ser eficaz para la aceptabilidad y adhesión de la PrEP. Los relatos de los adolescentes mostraron que las estrategias promovieron la prevención combinada del VIH, posibilitaron reuniones de reclutamiento, ayudaron a negociar y convencer a las personas a usar la PrEP, fortalecieron la educación entre pares y evocaron un sentimiento de “estar con” y “caminar juntos”, a pesar de los desafíos. Las interacciones, ya sean de manera presencial o *online*, con el uso de las tecnologías sociales, tuvieron un papel fundamental en el reclutamiento de adolescentes para el proyecto, en la ampliación del conocimiento sobre la PrEP y las demás estrategias de prevención combinadas, y en el acceso a servicios de salud y autocuidado.

## Introduction

The global response to HIV/AIDS should prioritize young people ^1^ ^,^ ^2^ given the rise in infections. Innovative strategies should be developed to broadly offer HIV pre-exposure prophylaxis (PrEP) combination prevention, especially to key youth populations ^3^ ^,^ ^4^ ^,^ ^5^ ^,^ ^6^.

The youth has multiple physiological, behavioral, and psychosocial specificities ^7^. Young people in the age groups from 15 to 19 and from 20 to 25 years show increasing HIV infection and AIDS diagnosis rates ^8^ ^,^ ^9^. However, protection and prevention actions in this group have indicated the need for expansion, a situation that became even more complex during the COVID-19 pandemic ^10^. Moreover, most PrEP demand generation actions among young people focused on prevention, have failed to use diverse and innovative educational approaches regarding didactic and methodological resources and access to the public in spaces of increased affective sociability ^11^ ^,^ ^12^.

Services and programs should implement demand creation for PrEP use to expand HIV combination prevention and other sexually transmitted infections (STIs). Innovative strategies would enhance PrEP acceptability and intent to use ^10^ among adolescents and young men who have sex with men (MSM) and transgender women (TGW) ^11^. Moreover, these actions may be necessary to promote healthcare by facilitating access to the health system, establishing bonds, strengthening networks, disseminating information, and increasing knowledge about HIV prevention ^12^.

Oral PrEP is a safe and effective prevention of HIV and should be scaled up among the sexually active youth and adolescents from key populations ^3^. Moreover, innovative approaches to create demand for PrEP use and prevention of other STIs should be developed ^13^ ^,^ ^14^ ^,^ ^15^ ^,^ ^16^. Thus, this study aimed to analyze the specificities and challenges of demand creation for the recruitment and participation of adolescent MSM and TGW (AMSM and ATGW, respectively) aged 15 to 19 years in a PrEP demonstration project in Brazil.

## Methods

A qualitative study was carried out from 2018 to 2021 as part of PrEP1519, the first demonstration cohort study of the effectiveness of daily oral PrEP among AMSM and ATGW in three Brazilian state capitals: Salvador (Bahia State), Belo Horizonte (Minas Gerais State), and São Paulo ^17^ ^,^ ^18^ ^,^ ^19^. Adolescents aged 15 to 19 years who identified as MSM or TGW were at increased risk of HIV infection. Those who reported living in the municipalities or their metropolitan areas in the studied area were eligible for the PrEP1519 cohort study. In this qualitative study, a purposive sample of 27 participants was recruited from the Salvador and São Paulo study sites.

We acknowledge that the adolescence and youth category is often considered in the health field as an essential biological dimension within fixed chronological limits that tend to naturalize, homogenize, and universalize subjects ^20^. Moreover, we highlight the importance of considering adolescents’ behaviors and their historical-cultural, symbolic, and lived dimensions. Moreover, the adolescent category is related to everyday practices regarded as socially representative of individuals considered young in a given cultural matrix ^21^. Criteria such as socializing in online/offline spaces; showing specific leisure, sports, and music preferences; using communication technologies; among others, may indicate the mosaic that defines youth in a given society.

Demand creation strategies ^3^ for PrEP recruitment were developed by the team based on a literature review, scientific consensus, and PrEP1519 group meetings to plan proposals the following discussions with AMSM and ATGW during and after formative research. Previous formative research was conducted to investigate the dynamics of social interaction, sexual sociability experiences, and acceptability of HIV prevention methods, including PrEP. Formative research used qualitative techniques with the observation of social spaces, in-depth interviews, and focus groups discussion with key informants.

**Figure 1:**
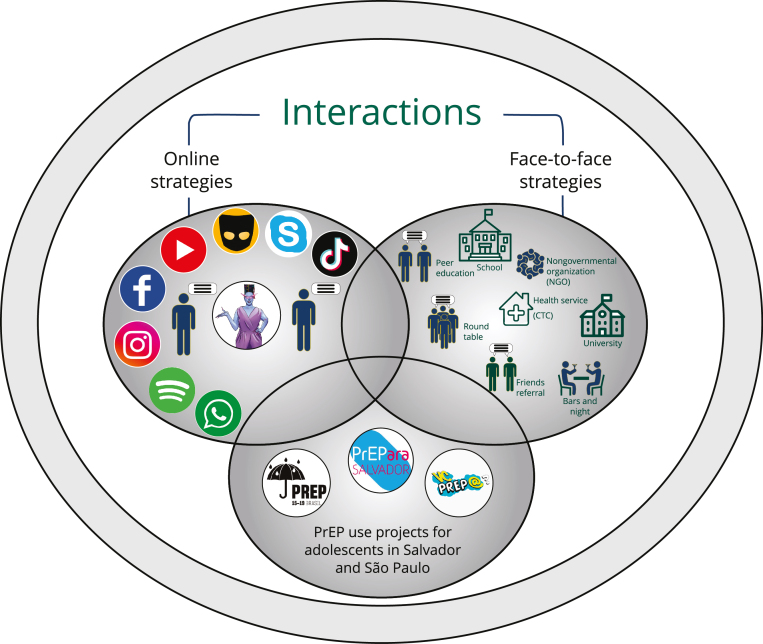
Exploratory model of demand creation for pre-exposure prophylaxis (PrEP) among adolescents based on interaction characterization of research participants. PrEP1519, June 2019-June 2020.

Demand creation strategies included online (virtual platforms) and face-to-face moments: (i) online: trained peer educators working on dating apps (Grindr, Tinder, Badoo, and Scruff, among others); trained peer educators working on social media (WhatsApp, Instagram, Twitter, and TikTok); and Amanda Selfie, the first transgender artificial intelligence chatbot in Latin America, which addressed HIV prevention and PrEP issues among young people, was developed on Facebook. Peer educators informed about Amanda Selfie on social media platforms. Young people interacted with her on Facebook messenger 24 hours per day/7 days per week, talked about sex, gender identity, HIV/STI prevention, and PrEP ^18^; and (ii) face-to-face: trained peer educators actions on HIV prevention in schools, youth meeting places, parties, and nongovernmental organizations (NGOs); referral by partner NGOs previously identified by the project; and referral by health services previously guided by the project ^19^ (Figure 1).

CTC: Counseling and Testing Center.

Recruited participants could choose one of two research arms to participate in this study: (a) the PrEP arm included those that enrolled in the daily use of oral PrEP with the antiretroviral tenofovir disoproxil fumarate (TDF), and emtricitabine (FTC) [TDF/FTC] combination; (b) the non-PrEP arm included those that were PrEP-eligible but chose, rather than using drug prophylaxis, to receive other HIV combination prevention methods (e.g., counseling, condoms, lubricant, douche, HIV post-exposure prophylaxis, and HIV self-testing) ^17^. The following selection criteria for the intentional sample of participants were adopted: having access to the project presentation, self-identified as a man who has sex with men and/or gay and/or transgender girls and having been nominated by peer educators and/or by interaction with Amanda Selfie in the virtual environment. We also looked for participants who discontinued the use of PrEP with the support of searches on digital social networks and peer educators.

The health team facilitated invitation for participation in this qualitative investigation, and interviewers were trained. Data were collected from June 2019 to June 2020. Researchers then established contact to arrange the best place and type of interview (in-person or remote). The 27 in-depth individual interviews were audio-recorded, namely: 11 in Salvador and 16 in São Paulo. In Salvador, the face-to-face interviews were held in private rooms at the PrEP clinic at a Diversity Center for the LGBTQIA+ community, which integrates a series of initiatives to promote and protect human rights, including the Bahia State Center for the Promotion and Defense of LGBTQIA+ Rights ^19^. In São Paulo, most interviews were held in a private room at University of São Paulo or places chosen by participants (e.g., NGO or in participants’ homes). Due to the COVID-19 pandemic, five interviews in São Paulo were conducted online via video conferencing or by smartphone.

The interviews were conducted using a semi-structured script with closed-ended questions related to participants’ sociodemographic and health characteristics and open-ended questions about the empirical object of this study, namely strategies and motivations that led young people to participate in the study, including questions about demand creation strategies, such as the perception of the adopted recruitment strategies, the performance of peer educators and health teams, barriers and facilitators of recruitment strategies, perception of approach types, among other topics related to the dynamics of their participation in the project and enrollment in a PrEP program. The interviews lasted a minimum of 30 minutes and a maximum of 90 minutes.

The interviews were fully transcribed, and the data were organized and systematized based on a coding process on NVivo, version 12 (https://www.qsrinternational.com/nvivo/home), which enabled the generation of thematic codes for the narratives. Subsequently, responses were subjected to Braun & Clarke ^22^ reflexive thematic content analysis by carefully reading the material, formulating new units of analysis, and identifying new themes, patterns, and subcategories. This process of analysis involved several cycles of increasing theoretical depth and reflexivity. Lastly, the research team internally validated the codes by consensus among the Salvador and São Paulo researchers. The data were interpreted based on the specific literature. We highlight the importance of understanding narratives and stories about HIV/AIDS and their prevention perspectives under a sociocultural-oriented approach by dialogically acknowledging that personal and cultural narratives are interconnected ^23^.

This study was performed in accordance with ethical aspects and complied with the recommendations of the legislation on research involving human beings in Brazil. The research project was approved by the Research Ethics Committee of the World Health Organization (WHO; protocol ID: Fiotec-PrEP Adolescent Study) and by the local Ethics Committees in Brazil, namely those of the Faculty of Medicine, University of São Paulo (FMUSP; protocol n. 70798017.3.0000.0065) and the Institute of Public Health, Federal University of Bahia (UFBA; protocol n. 3.224.384).

All 18- and 19-year-olds signed an informed consent form, providing their consent to participate in this study and the of use their data for scientific purposes. Several protocols were applied to adolescents younger than 18 years in each state in accordance with the considerations of the respective Ethics Committees. In Salvador, the following procedures were adopted: (a) informed consent form, which was signed by a parent or guardian and the assent form for minors, which was signed by the adolescent; (b) an assent form signed by the teenager when the psychologist and the social worker of the research team assessed that the adolescents’ family ties were broken or that they were at risk of suffering physical, psychological, or moral violence for their sexual orientation. In São Paulo, the following procedure was followed: (a) a court decision waiving parental consent, which explains the reason for the informed consent form being signed by the adolescents sufficed for joining this study.

To ensure anonymity, participants were identified by their code name: Y - young, followed by the participation order number, age, gender identity, sexual orientation, guardian, or means of recruitment and the municipality and state of residence.

## Results

Most participants were young cisgender MSM and transgender women aged 16 to 20 years, had schooling within the expected range for their age (attending secondary education or starting higher education), and self-reported as black or mixed-race. Various PrEP demand creation strategies were used, expanding the possibilities of contact and recruitment for the study (Box 1), namely by an NGO (two participants), face-to-face peer educators (nine participants), online peer educators (six participants), health service referral (five participants), referral from friends and affective-sexual partners (three participants), and Amanda Selfie (one participant). Most participants (18) opted to enroll in the PrEP.

**Box 1:** Characterization of research participants. PrEP1519, June 2019-June 2020.

NUMBER	AGE	GENDER IDENTITY	SEXUAL ORIENTATION/ RELATIONSHIPS	ETHNICITY/SKIN COLOR	EDUCATION	CITY OF RESIDENCE	DEMAND CREATION STRATEGY	ENROLLMENT IN THE PrEP1519 PROJECT	ENROLLMENT IN THE PrEP 1519 PROJECT AND IN PrEP
1	20 years	Transgender woman	Bisexual and asexual	White	Complete secondary education	São Paulo	NGO	Yes	Yes
2	17 years	Cisgender man	MSM	White	Complete secondary education	São Paulo	Health service (CTC)	No	N/A
3	19 years	Transgender woman	Bisexual	White	Incomplete higher education	Salvador	Peer education in a political movement	Yes	Yes
4	18 years	Cisgender man	MSM	N/A	N/A	São Paulo	Online peer education (Grindr)	No	N/A
5	18 years	Cisgender man	MSM	N/A	Complete secondary education	São Paulo	Online peer education (Grindr)	No	N/A
6	18 years	Cisgender man	Homosexual	Mixed-race	Incomplete secondary education	Salvador	Online peer education (Instagram)	No	N/A
7	N/A	Transgender woman	N/A	N/A	N/A	São Paulo	Peer education in social spaces	Yes	Yes
8	19 years	Transgender woman	Heterosexual	Mixed-race	Incomplete higher education	Salvador	Referral by friend	Yes	No
9	22 years	Cisgender man	N/A	N/A	N/A	São Paulo	NGO	No	N/A
10	19 years	Non-binary	Pansexual	Mixed-race	Incomplete higher education	Salvador	Peer education in social spaces	Yes	Yes
11	19 years	Transgender woman	Heterosexual	Indigenous	Incomplete primary education	São Paulo	Health service (CTC)	Yes	Yes
12	18 years	Transgender woman	Heterosexual	White	Complete seconday education	São Paulo	Health service (CTC)	Yes	Yes
13	18 years	Cisgender man	Homosexual	Mixed-race	Complete secondary education	Salvador	Online peer education (Grindr)	Yes	No
14	19 years	Transgender woman	Pansexual	White	Attending higher education	São Paulo	Peer education in social spaces	Yes	Yes
15	18 years	Gender fluid	Pansexual	Mixed-race	Complete secondary education	São Paulo	Peer education in social spaces	Yes	Yes
16	19 years	Cisgender man	Homosexual	Mixed-race	Incomplete higher education	Salvador	Referral by friend	Yes	Yes
17	20 years	Transgender woman	Heterosexual	Mixed-race	Complete secondary education	São Paulo	Peer education in social spaces	Yes	Yes
18	18 years	Cisgender man	Homosexual	Mixed-race	Incomplete higher education	Salvador	Peer education in social spaces	Yes	Yes
19	17 years	Cisgender man	Homosexual	Black	Incomplete secondary education	Salvador	Online peer education (Grindr and WhatsApp)	Yes	Yes
20	17 years	Transgender woman	N/A	White	Incomplete secondary education	São Paulo	Health service (CTC)	Yes	No
21	17 years	Cisgender man	Homosexual	Mixed-race	Attending secondary education	São Paulo	Peer education in social spaces	Yes	Yes
22	17 years	Cisgender man	Pansexual	Black	Attending secondary education	São Paulo	Chatbot Amanda Selfie	Yes	Yes
23	18 years	Cisgender man	Homosexual	Black	Complete secondary education	São Paulo	Health service (CTC)	Yes	Yes
24	19 years	Cisgender man	Homosexual	White	Attending higher education	São Paulo	Online peer education (Grindr)	Yes	Yes
25	19 years	Transgender woman	Heterosexual	Black	Complete secondary education	Salvador	Referral by friend	Yes	Yes
26	18 years	Cisgender man	Bisexual	Black	Complete secondary education	Salvador	Referral by friends and sexual partners who participated in the project	Yes	No
27	18 years	Transgender woman	Heterosexual	White	Complete secondary education	Salvador	Peer education in social spaces	Yes	Yes

CTC: Counseling and Testing Center; MSM: men who have sex with men; N/A: not available; NGO: nongovernmental organization; PrEP: pre-exposure prophylaxis.

Participants’ demand creation strategies for enrolling in the PrEP1519 study highlight interactions and bonding in face-to-face strategies and socio-affective spaces mediated by digital platforms. Moreover, their narratives explained in particular the specificities, limits, and challenges of recruiting a young population, especially for those young people who chose to not use PrEP.

In Salvador, the first interactions in which volunteers participants occurred in activities held in youth venues, such as sexual health workshops and demand creation strategies aimed at combination prevention conducted by the PrEP1519 team in night clubs, parties, and meetings of organized social movements and schools. Moreover, in São Paulo, young users of CTC (Counseling and Testing Center) Henfil initially showed a spontaneous demand for HIV testing, which was followed by PrEP demand creation strategies by healthcare providers.

### Recruitment challenges, PrEP knowledge, social context, and living conditions of young people

Although participants positively evaluated demand creation strategies, some adolescents reported low awareness and doubts about PrEP. Moreover, they reported feeling insecure about participating in the project, especially due to unclear information about PrEP during some approaches, fear of taking medication, and the context of the COVID-19 pandemic.

“...*at the time of recruitment, the project team was concerned about the sexual health issues of transgender people. They talked and gave a lot of guidance. Moreover, they answered my questions. However, I faced some difficulties (such as lack of time) to find all the information and the need to comply with the quarantine given the risk of COVID-19, and I did not have the courage to tell my family about participating in the project*” (A/J-09, 22-year-old, cisgender man, avoided reporting his sexual orientation, recruited by a recruiter but did not join the project, São Paulo).

Building dialogic relationships based on mutual respect with adolescents has helped to expand PrEP demand creation and other HIV combination prevention strategies. These strategies were perceived as innovative, promoting empathy and stimulating health care self-perception.

“...*I was at a party when I was approached and answered a survey. Soon after, I was invited to participate in the PrEP project* (...) *it was unusual. At first, I was in disbelief because I was in a festive environment, but then I found it very cool and positive because it is usually a place where healthcare professionals do not go, but peer educators were there recruiting and bringing information about PrEP to people, most of whom are unaware of it*” (A/J-03, 19-year-old, TGW, bisexual, recruited by a peer educator, Salvador).

Generally, ATGW positively evaluated recruitment activities and highlighted concerns about the gender transition phase (e.g., drug interaction between PrEP and hormones). Moreover, the brief recruitment strategy, i.e., handing out an information leaflet on the street, following up with a telephone call, and accompanying participants in person from the place of transport to the end of care at the clinic was considered innovative by a transgender girl.

“...*I chose to participate in the project, which has a very positive approach to recruitment, improving my understanding of HIV prevention and PrEP, but I chose not to use PrEP because I already use other medications, such as feminizing hormone therapy*” (A/J-08, 19-year-old, TGW, heterosexual, recruited by a friend/peer education who did not use PrEP, Salvador).

“...*I was recruited on the street at downtown São Paulo, and they gave me an information leaflet about PrEP and the project. They provided brief information, wrote down my name and phone number, and then called me* (...) *incentivizing me, and I decided to participate. To get to the project unit, the recruiter met me at the subway station, took me to the project unit and accompanied me until I completed the service. It was a very different and innovative treatment*” (A/J-07, TGW, not report her age or sexual orientation, recruited by a recruiter, São Paulo).

Adolescents are affected by different factors that influence their enrollment in the project. For example, HIV/AIDS-related stigma was associated with PrEP in the context of interpersonal relationships between friends and sexual partners, which led a participant to lie about using this preventive resource. Nevertheless, the project was also reported as a meeting space for peers who shared similar experiences, contributing to engagement in social activism and adherence to preventive care, thereby raising interest in and bringing different perspectives for interactions beyond HIV prevention.

“...*Although the project is positive, it is not easy to join because society is hypocritical and generates a lot of stigma, which forces people to lie about PrEP; they do not want to be associated with the risk group. In addition, I experienced pressure from friends and people I had sex with not to use PrEP*...”. (A/J-15, 18-year-old, gender-fluid person, pansexual, recruited by a recruiter, São Paulo).

### Online demand creation: knowledge production, self-identification, and estrangement

The knowledge participants reported about PrEP was generally acquired by browsing the internet. In this context, a participant’s narrative highlighted that he had “noticed the subject of PrEP” (perhaps for the first time) on the profile of a user who reported using this method on a dating app. Some relationship apps and/or sexual encounters enable users to post the result of their HIV test and the use of PrEP in their profiles, as shown below.

“...*Once, while using Grindr, I saw some profiles with the description: ‘negative, using PrEP’. Since then, I started noticing this matter. Soon after, I saw a profile with the PrEP logo; it was the profile of the PrEP project in Salvador. I found it unusual and very innovative*” (A/J-10, 19-year-old, non-binary person, pansexual, recruited by a peer educator, Salvador).

The customized LGBTQIA+-friendly project profiles on social media and dating apps made it possible to reach out to, self-identify with, and spread knowledge about HIV testing, combination prevention, and PrEP among young people. Additionally, participants highlighted the posture of peer educators in the interaction as welcoming and relaxed, perhaps contrasting with the traditional approach of healthcare providers.

“...*It is great to use dating apps to recruit people for the project because many gay men use them to hook up* (...)*. It is a way of helping young people to get to know each other and to know how to better deal with sexual intercourse. I thought it was amazing*” (A/J-13, 18-year-old, cisgender male, homosexual, recruited by a peer educator on Grindr, Salvador).

“...*I was invited to participate in the project while I was using Grindr. They gave me initial information, which was provided in more detail when I visited the service facility. It was stimulating, to the point where I called some friends, told them what had happened and invited them to participate with me in the project*...”. (A/J-24, 19-year-old, cisgender man, homosexual, recruited by a recruiter on Grindr, São Paulo).

Although these online strategies opened up numerous opportunities for interacting with these groups more often, some participants reported feeling strange and insecure. The credibility of the message offered by non-institutional profiles was the main issue.

“...*I received a message on Grindr asking about my PrEP use and sex life. From then on, I received an invitation offering access to PrEP and participation in the project. I did not expect to be approached with such a subject in the application. I found the interaction very important and valid, but it felt kind of weird and I was insecure because it was not an institutional profile, but an individual profile*” (A/J-11, 19-year-old, TGW, heterosexual, recruited by recruiters, São Paulo).

The demand creation strategies for PrEP use in the project involved a technological innovation: Amanda Selfie, the first TGW chatbot in Latin America, designed to answer questions about sex, sexuality, sexual orientation, gender identity, sexual practices, and culture of the LGBTQIA+ community. Amanda Selfie also played a key role in the project, identifying risk behavior to HIV and other STIs. She expanded access to health services available in the healthcare system. She promoted the self-identification of young people with the project by using a simple and visual language of a character who draws Facebook Messenger users’ attention and enables interactions with online access 24 hours a day.

The narratives that explain the role of Amanda Selfie in PrEP demand creation are illustrated below; two narratives stand out, namely that of a gender-fluid young woman suggesting that transgender people identify with the image of Amanda and thus with this approach strategy for participation in the project and the narrative of a young cis man who also identified with the Amanda Selfie strategy.

“...*I was recruited through social media, through ‘Amanda Selfie’, a robot, which I considered very interesting, creative, and valuable for finding people and publicizing the project* (...)*. It was an important strategy, which helped and encouraged me to use PrEP. I liked the strategy so much that I decided to stay on the project*” (A/J-15, 18-year-old, gender fluid, pansexual, recruited by a recruiter, São Paulo).

### Peer education as a networking promoter to overcome barriers and difficulties

The demand creation for PrEP use with MSM and TGW peer educators and other mediators from the same community created a network in the sense of “being with the other”, “being part of”, and “being like the other” (Figure 1). It was based on communicative interaction to enable networking with the key population, thereby overcoming barriers and difficulties in learning about and accessing the project.

In line with the above, peer education plays a crucial role in the decision-making process of participation in the project, standing out as a PrEP demand creation agent among adolescents and helping participants acquire a sense of belonging and proximity to the other. Thus, from these belonging and proximity spaces, peer education also plays a vital role in the healthcare process of these young people.

“...*The project offered me so much more than PrEP. Recruitment through peer educators allowed me to have access to condoms, lubricant, douches, access to information and health education, assistance with specialized professionals to assist trans and TGW people* (...)*. Today, I feel safer to deal with my own body*” (A/J-17, 20-year-old, TGW, heterosexual, recruited by a peer educator, São Paulo).

“...*I was at a dance and there were recruiters publicizing the project. They came to talk to me and I agreed to given them my telephone number so that they could talk to me a little more about the project. They sent a message via WhatsApp and then they called me. I agreed to visit the service. I really liked the strategy used in the approach, and that made me decide to participate*” (A/J-21, 17-year-old, cisgender man, homosexualrecruited by a peer educator, São Paulo).

## Discussion

This study showed that the demand creation strategies for the participation of young people in PrEP1519 were essential for mediating young people and the project and establishing and strengthening affective bonds, which may satisfactorily and positively influence the decision-making processes for acceptability and bonding to PrEP. Adolescents’ narratives showed that demand creation strategies disseminated HIV combination prevention, opened recruitment meeting opportunities, and helped negotiate with and support individuals to use PrEP. These strategies, strengthened by peer education, evoked a feeling of “being with” and “walking together” despite challenges, such as fear, insecurity, and online risks.

All recruitment/capture actions were configured around three important activities: (1) distribution of materials and supplies (condoms, lubricants, self-test kits, etc.); (2) discursive approaches around HIV/AIDS prevention; and (3) publicizing the PrEPara Salvador clinic space (including publicizing and encouraging the use of PrEP, combined prevention, and other services, such as testing and multidisciplinary counseling).

The development of a set of strategies for hybrid recruitment constituted an innovation for recruiting adolescents/young people to use PrEP. In this context, a prior survey of the territory, the profile of participants, forms of socialization, access to digital resources, the creation of a specific system for monitoring the use of PrEP by participants in different municipalities (SisPrEP), human involvement, and a robot contributed to achieving diversity and innovation.

Strategies aimed at fighting the HIV epidemic have been crucial for the quality of life of the global population. Adopting prevention measures, strengthening health promotion and education, raising health literacy ^24^ ^,^ ^25^, and overcoming barriers related to stigma constitute significant challenges to be overcome and include technological advances that respect sociocultural specificities ^3^ ^,^ ^4^ ^,^ ^5^ ^,^ ^6^. An example of this was the use of robotization, based on the creation of the Trans Robot Amanda Selfie, which was created by an artificial intelligence project to elucidate doubts about sexuality and prevention and treatment of sexually transmitted infections.

Overcoming obstacles to the participation of adolescents and young people (whose age and generation specificities offer key complicating factors for vulnerability to HIV infection) in HIV prevention programs and policies further increases the importance of demand creation strategies in this scenario. Strengthened actions are still needed ^9^ ^,^ ^15^ ^,^ ^16^ ^,^ ^26^ ^,^ ^27^.

The gap in scientific knowledge on the potential and complicating factors in PrEP and demand creation among young people must be bridged ^10^ ^,^ ^13^ ^,^ ^14^. This problem is worsened when such factors intertwine with social markers of difference, intersectionality, and social and health vulnerabilities, as in the LGBTQIA+ population ^28^ ^,^ ^29^. This population is strongly affected by the denial of social and health rights, facing barriers to access HIV/AIDS care, prevention, and treatment technologies, which impact PrEP adherence ^30^ ^,^ ^31^.

Furthermore, results highlighted the social interaction elements necessary for understanding young people when recruiting them for such a project aimed at showing PrEP use, which may explain unknown aspects regarding the meanings of recruitment as a critical moment for knowledge and the decision to use PrEP. Considering our results, this approach must be further explored ^32^ ^,^ ^33^ ^,^ ^34^ ^,^ ^35^.

The recruitment team attended LGBTQIA+ parties and tourist attractions in the municipalities. Moreover, partnerships were established with public schools to hold conversation circles and games to inform the youth about combined prevention strategies to prevent HIV and publicize the PrEP use project.

Other studies have shown that people tend to adhere to educational interventions ^30^ focused on prevention throughout their life cycle. Health education configures a valuable and powerful device to enhance knowledge, raise awareness of health risks, control and promote them among individuals, and prevent diseases ^36^ ^,^ ^37^ ^,^ ^38^.

Reaching out to adolescents and including them in HIV prevention programs may entail limiting aspects, such as schooling, adolescence, and behavioral and psycho-emotional difficulties, which may significantly negatively affect their affective-sexual relationships when impacted by sexual and gender norms ^39^ ^,^ ^40^ ^,^ ^41^ ^,^ ^42^ ^,^ ^43^ ^,^ ^44^.

Our findings indicated that an empathic, interactive, and self-identification relationship of being “among peers” is more critical for demand creation actions than their approach or design ^19^.

Following young people in their participation in a PrEP demonstration project will require an expanded territorial exploration of their places of residence and demand creation strategies and resources. Peer education, interaction, and communication processes can produce satisfactory results regarding acceptability and adherence to projects with this purpose ^39^ ^,^ ^40^. It is believed that the generation of demands for the use of PrEP based on a recruitment strategy with peer educators stemmed from the proximity between participants’ and recruiters’ cultural, social, and language aspects and the identification with sexual and gender identities recognized by the teenage audience reached with the project.

Coincidentally, most peer educators had some identity trait affinity, which may have provided an opening for the narrative of experiences and the establishment of a bond, a relationship of trust, reduction of fears, and the establishment of dialogue and encouragement for participation in the project and the use of PrEP.

PrEP1519 developed several demand creation strategies to inform adolescents about PrEP, recruit them for its use, and facilitate their enrollment in PrEP services ^45^. Thus, focusing on the possibilities of social interactions in these strategies may mitigate the negative impacts on the health of young people in contexts of vulnerability to HIV infection and help to expand healthcare to this population ^45^.

The limitations of this study include the impossibility for a more detailed analysis of differences between groups (e.g., young transgender women and cisgender men), approaches (recruitment on the streets or via NGOs, apps, and services), and the time participants arrived or joined the project. New studies should be conducted to further delve into the dimensions of vulnerability (especially of young transgender women) and differences between groups and approaches in the impact on PrEP demand creation, including considering online demand creation strategies, such as using apps and chatbots.

## Conclusion

Whether face-to-face or online via social media platforms, interactions promoted by youth recruitment strategies for their participation in a PrEP demonstration project adhered to these young people’s life contexts and experiences. These demand-creation strategies motivated, attracted, and raised young people’s awareness in two different scenarios in Brazil, producing meanings about recruitment and transposing them to other life and health dimensions.

The strategies in this study significantly expanded knowledge about PrEP and other combination prevention strategies regarding self-care and access to health services among young people. These strategies evoked feelings of being among peers, thus improving responses to recruitment and demand-creation processes. Such an approach positively influenced the interaction and mediation of young people with the project and established and strengthened bonds, especially contributing to PrEP acceptance and use of decision-making processes, as shown in this study.
